# Exploring the Potential of a Wearable Camera to Examine the Early Obesogenic Home Environment: Comparison of SenseCam Images to the Home Environment Interview

**DOI:** 10.2196/jmir.7748

**Published:** 2017-10-12

**Authors:** Stephanie Schrempft, Cornelia HM van Jaarsveld, Abigail Fisher

**Affiliations:** ^1^ Department of Behavioural Science and Health University College London London United Kingdom; ^2^ Department for Health Evidence Radboud University Medical Center Nijmegen Netherlands; ^3^ Department of Primary and Community Care Radboud University Medical Center Nijmegen Netherlands

**Keywords:** environment and public health, obesity, parents

## Abstract

**Background:**

The obesogenic home environment is usually examined via self-report, and objective measures are required.

**Objective:**

This study explored whether the wearable camera SenseCam can be used to examine the early obesogenic home environment and whether it is useful for validation of self-report measures.

**Methods:**

A total of 15 primary caregivers of young children (mean age of child 4 years) completed the Home Environment Interview (HEI). Around 12 days after the HEI, participants wore the SenseCam at home for 4 days. A semistructured interview assessed participants’ experience of wearing the SenseCam. Intraclass correlation coefficients (ICCs), percent agreement, and kappa statistics were used as validity estimates for 54 home environment features.

**Results:**

Wearing the SenseCam was generally acceptable to those who participated. The SenseCam captured all 54 HEI features but with varying detail; 36 features (67%) had satisfactory validity (ICC or kappa ≥0.40; percent agreement ≥80 where kappa could not be calculated). Validity was good or excellent (ICC or kappa ≥0.60) for fresh fruit and vegetable availability, fresh vegetable variety, display of food and drink (except sweet snacks), family meals, child eating lunch or dinner while watching TV, garden and play equipment, the number of TVs and DVD players, and media equipment in the child’s bedroom. Validity was poor (ICC or kappa <0.40) for tinned and frozen vegetable availability and variety, and sweet snack availability.

**Conclusions:**

The SenseCam has the potential to objectively examine and validate multiple aspects of the obesogenic home environment. Further research should aim to replicate the findings in a larger, representative sample.

## Introduction

The home environment is thought to play an important role in early obesity prevention and weight management [1-3]. Researchers have identified food, physical activity, and media-related influences as core domains that define the obesogenic home environment [4]. Multiple self-report measures have been used to examine aspects within home environment domains, but few are comprehensive, and few have been assessed in terms of criterion validity (the extent to which they relate to concrete criteria in the real world) [5]. The Home Environment Interview (HEI) is one of few comprehensive home environment measures and has recently been associated with diet, physical activity, and TV viewing in a large sample of preschool children [6].

Demonstrating the criterion validity of parent- or self-reported measures (which are prone to social desirability and recall biases) is important to ensure that the results of studies using them are largely unattributable to measurement error. In the case of the home environment, identifying accurate associations with health behaviors and/or weight is important for ensuring the design of effective weight-related interventions. Studies that have assessed criterion validity have tended to use one-off home visits that cannot capture behavioral or social aspects of the home environment, such as mealtime interactions and parental modeling [7]. Multiple home visits can provide further insight [8], but they are costly and labor intensive.

Technologic advances have provided opportunities to objectively examine the obesogenic home environment. Video recording has long been used by developmental researchers to assess child-parent interactions, including those at mealtimes [9-11]. Disposable cameras have been used to capture the food environment from the child’s perspective [12]. Although insightful, standard picture cameras do not permit continuous recording and video cameras do not capture events from the first-person perspective, which would provide a more detailed and naturalistic account of an individual’s environment.

Visual lifelogging refers to the passive digital capture of everyday activities from the first-person perspective. Numerous devices have been developed for visual lifelogging [13]. The most popular wearable camera in a research setting is the SenseCam (Microsoft Corp) [14], designed to take pictures automatically (approximately every 20 seconds) when triggered by sensors that log temperature, light, acceleration, and passive infrared data [15]. The SenseCam is straightforward to use, has a long battery life (up to 16 hours), a large storage capacity (over one week’s worth of images), a wide-angle lens to capture everything within the wearer’s view, and does not record sound [16]. Each image is time-stamped so duration of specific events or activities can be deduced.

The SenseCam has predominantly been used in memory and cognitive impairment research [17,18]. More recent research has explored how the SenseCam can be used to assess diet and activity behaviors. SenseCam images have been compared with travel diaries in volunteer adults [19] and teenagers [20], food diaries [21], 24-hour dietary recall [22], and accelerometers in university employees to improve the classification of sedentary behavior [23], highlighting the utility of a wearable camera to validate traditional assessment tools. The SenseCam has also been used to examine the context of eating behavior in adult [24] and teenage [25] participants. No studies have used a wearable camera to examine the early obesogenic home environment.

This study will therefore examine whether the wearable camera SenseCam can be used to examine the early obesogenic home environment and whether it is useful for validation of self-report measures. Specifically, this study will examine whether the Sensecam is acceptable to participants, which aspects of the obesogenic home environment can be captured by the SenseCam, and how this information compares to that captured by the HEI [6].

## Methods

### Study Sample

The study sample was obtained using convenience sampling. Participants were 15 parents of children aged 2 to 8 years who had taken part in previous research at University College London and agreed to be contacted for future studies. A total of 94 parents were sent an invitation letter. Parents who did not respond to the letter were followed up with a telephone call. Participants gave written consent before taking part. Any other adults living in the home also consented to participation, since they would be photographed. Ethical approval for the study was granted by the University College London Ethics Committee for Research Involving Human Subjects (project approval number 3792/001). The study protocol adhered to the ethical framework outlined by Kelly and colleagues [26].

### Measuring and Validating the Home Environment

Participants completed the HEI by telephone while at home. The HEI is one of few comprehensive measures of the home environment, capturing multiple aspects of the food, physical activity, and media domains. Items assess food availability and accessibility, physical activity opportunities, and media equipment availability, as well as social aspects such as parental modeling of eating and activity behaviors. The HEI was adapted from the Healthy Home Survey [7], the most comprehensive home environment measure available at the time, and with evidence for criterion validity [7]. Consistent with the Healthy Home Survey, the test-retest reliability of the HEI (assessed in a sample of 44 parents) was generally moderate to high. The intraclass correlation coefficients (ICCs) and 95% confidence intervals for the total scores were as follows: food environment (0.71, 0.52-0.83), activity environment (0.83, 0.72-0.91), media environment (0.92, 0.85-0.95), and overall (0.92, 0.86-0.96). Additional details of the HEI are provided in a previous publication [6].

Participants were visited at home on average 12 (SD 5.82) days after completing the HEI. The time frame between completing the HEI and wearing the camera was chosen to be largely consistent with the validation study of the Healthy Home Survey, where the home visit took place 7 to 14 days after the initial telephone interview. Participants were asked to wear the camera during waking hours while at home for 4 consecutive days (including at least one weekend day). A 4-day wearing period was chosen to strike a balance between capturing sufficient information about the home environment for the purposes of the study and minimizing participant burden. Participants wore the SenseCam on a lanyard around their neck with adhesive fashion tape attached to the back to reduce movement. Participants were told that they were free to turn off or remove the camera whenever they did not feel comfortable wearing it. The following statement was provided for participants to use if they encountered other people while wearing the camera: “I am volunteering for a research project looking at my home environment. The device is called SenseCam and it takes pictures of my daily activities.” Previous research has found that this approach is sufficient to satisfy any queries from other members of the public [19].

### Semistructured Interview

After the wearing period, the camera was collected and participants completed a semistructured interview. Participants were asked about ease of use, awareness of the camera, reactions from other people, instances where they did not feel comfortable wearing the camera, and whether they felt that wearing the SenseCam could influence families to change aspects of their household routine. Participants had the opportunity to view and delete their images if they did not wish to have them stored for analysis.

### Statistical Analysis

The SenseCam images were manually coded using the Oxford CLARITY-DCU SenseCam browser [27]. Each image was visually inspected and coded for the presence or absence of features assessed in the HEI. Home environment features that could not be captured by the SenseCam were identified before coding and included whether the child was allowed to help him or herself to food and drink; the frequency the child was allowed to play inside and outside the home; parks and indoor recreation centers close to the home; and rules around media use. A total of 54 features were coded (42 food-, 2 activity-, and 10 media-related). These are shown in Tables 2 and 3 and in Multimedia Appendix 1 alongside the corresponding HEI questions.

Images were classified as uncodeable if there were low light levels, something was covering the lens, or in cases of extreme blurring. Home environment features were coded as missing if they were not identifiable in the images.

A total of 60 days of data (75,818 images) were coded. It took 100 hours to code the data. One randomly selected day’s worth of images was recoded by the original coder after study completion to assess intrarater reliability. For interrater reliability, an independent coder analyzed another randomly selected day’s worth of images. There was almost 100% agreement across coding sessions.

ICCs (for continuous variables), percent agreement, and kappa statistics (for categorical variables) were used as validity estimates. As recommended, kappas and ICCs were defined as: <0.40=poor, 0.40-0.59=fair, 0.60-0.74=good, and 0.75-1.00= excellent [28]. In cases where percent agreement was high (≥80) but kappa was poor, the proportion of positive (ppos) and negative (pneg) agreement were presented. This is recommended for better understanding of results [29].

## Results

### Study Sample

Of the 94 parents contacted, 34 (36%) did not respond to the initial letter and could not be contacted by telephone or email. Among those who responded and did not wish to participate in the study, 62% (28/45) cited discomfort with wearing the camera as the reason and 38% (17/45) cited other reasons such as lack of time. Participants included 13 mothers and 2 fathers. All were main caregivers of their children. Parent and child characteristics are shown in Table 1.

**Table 1:** Characteristics of families who took part in the study.

Characteristics			Mean (SD) or n (%)
**Parent characteristics**			
	Age (years), mean (SD)		38.6 (6.4)^a^
	**Education level^b^, n (%)**		
		Low	1 (7)
		Medium	2 (13)
		High	12 (80)
	**Ethnicity, n (%)**		
		White	13 (87)
		Other	2 (13)
	**Number of children in the home, n (%)**		
		One	5 (33)
		More than one	10 (67)
**Child characteristics**			
	Age (years), mean (SD)		4.8 (1.7)
	**Sex, n (%)**		
		Male	10 (67)
		Female	5 (33)
	**Ethnicity, n (%)**		
		White	9 (60)
		Other	6 (40)

^a^Data were missing for 1 participant on this variable (n=14).

^b^Education level categorized as low (no qualifications or basic high school education), medium (vocational or advanced high school education), and high (university-level education).

**Table 2:** Descriptive statistics for the home environment features (N=15; n [%] who responded yes or mean [SD]).

Home environment feature		HEI^a^	SenseCam
**Food availability, n (%)**			
	Fresh fruit	15 (100)	15 (100)
	Tinned fruit	6 (40)	0 (0)
	Dried fruit	9 (60)	4 (27)
	Frozen fruit	3 (20)	0 (0)
	Fresh vegetables	14 (93)	15 (100)
	Tinned vegetables	14 (93)	7 (47)
	Frozen vegetables	13 (87)	4 (27)
	Savory snacks	10 (67)	8 (53)
	Sweet snacks	12 (80)	6 (40)
	Confectionery	10 (67)	4 (27)
	Fruit juice	8 (53)	11 (73)
	Squash	5 (33)	4 (27)
	Fizzy drinks	2 (13)	4 (27)
	Smoothies	3 (20)	1 (7)
	Skimmed/semiskimmed milk	10 (67)	13 (87)
	Full-fat milk	5 (33)	6 (40)
**Food variety, mean (SD)**			
	Fresh fruit	3.5 (1.4)	4.5 (2.3)
	Tinned fruit	0.6 (0.9)	0 (0)
	Dried fruit	1.9 (1.9)	0.3 (0.6)
	Frozen fruit	0.2 (0.4)	0 (0)
	Fresh vegetables	6.3 (3.0)	6.7 (3.1)
	Tinned vegetables	3.9 (1.7)	0.8 (1.0)
	Frozen vegetables	1.7 (1.4)	0.3 (0.5)
	Savory snacks	1.1 (1.1)	0.7 (0.7)
	Sweet snacks	1.5 (1.1)	0.7 (1.1)
	Confectionery	0.9 (0.8)	0.3 (0.5)
**Food displayed, n (%)**			
	Any fruit	15 (100)	14 (93)
	Ready-to-eat vegetables	2 (13)	0 (0)
	Savory snacks	0 (0)	0 (0)
	Sweet snacks	3 (20)	2 (13)
	Confectionery	1 (7)	1 (7)
	Fruit juice	0 (0)	0 (0)
	Squash	2 (13)	3 (20)
	Fizzy drinks	1 (7)	0 (0)
	Smoothies	0 (0)	0 (0)
**Family meals, n (%)**			
	Breakfast	11 (73)	11 (73)^b^
	Lunch	12 (80)	10 (67)^c^
	Dinner	11 (73)	12 (80)^b^
**Child eating while watching TV, n (%)**			
	Breakfast	0 (0)	2 (13)^d^
	Lunch	0 (0)	0 (0)^e^
	Dinner	1 (7)	1 (7)^d^
	Snacks	5 (33)	2 (13)^f^
**Activity facilities, n (%)**			
	Garden	12 (80)	10 (67)
	Garden equipment	2 (17)	1 (8)^g^
**Household media equipment, mean (SD)**			
	Number of TVs	1.5 (1.1)	1.6 (1.1)
	Number of VCR/DVD players	1.5 (1.0)	1.3 (0.9)
	Number of computers	2.4 (1.0)	1.6 (0.9)
	Number of games consoles	0.7 (1.0)	0.2 (0.6)
	Presence of cable or satellite, n (%)	9 (60)	3 (20)^h^
**Child’s bedroom media equipment, n (%)**			
	TV	2 (13)	3 (20)^i^
	Computer	1 (7)	1 (7)^i^
	Console	2 (13)	1 (7)^i^
**Caregiver TV viewing (hours), mean (SD)**			
	Weekday	1.7 (1.3)	1.2 (0.7)^j^
	Weekend	2.4 (1.67)	1.5 (0.81)^k^

^a^HEI: Home Environment Interview.

^b^Two cases were coded as missing: 1 participant did not wear the SenseCam during breakfast time and 1 participant said during the semistructured interview that they had modified their mealtime routine.

^c^Three cases were coded as missing: 2 participants did not wear the SenseCam during lunchtime and 1 participant had modified their mealtime routine.

^d^Data were missing in 3 cases: 1 did not wear the SenseCam at breakfast/dinner time, 1 said in the semistructured interview that they had modified their mealtime routine, and the third did not have breakfast/dinner with their children during the wearing period.

^e^Data were missing in 5 cases: 2 did not wear the SenseCam at lunchtime, 1 said that they had modified their mealtime routine, and the last 2 did not have lunch with their children during the wearing period.

^f^Data were missing in 1 case where the caregiver did not wear the SenseCam around their child.

^g^Three cases were coded as missing as the garden wasn’t fully visible during the wearing period.

^h^It was only possible to determine the presence or absence of cable or satellite in 4 cases; the remaining cases were coded as missing.

^i^Two cases were coded as missing because the child’s bedroom was not visible during the wearing period.

^j^Data were missing in 6 cases where the caregiver did not wear the SenseCam for all of the weekday periods (morning/afternoon/evening).

^k^Data were missing in 7 cases where the caregiver did not wear the SenseCam for all of the weekend periods (morning/afternoon/evening).

### Measuring and Validating the Home Environment

**Figure 1:**
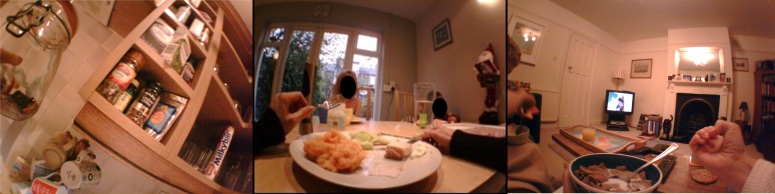
Sample SenseCam images showing the presence of confectionery (left), a family dinner (center), and eating breakfast while watching TV (right). Faces are colored for anonymity.

Participants wore the SenseCam for 4 (SD 1.1) days on average. The average wearing time per day was 5.9 (SD 2.6) hours *.* All 54 home environment features were captured to some extent. What was captured by the SenseCam depended on the duration of the wearing period and participant behavior during this period. As shown in Table 2, fresh fruit and vegetables were captured in all cases, tinned and frozen foods were rarely captured, and energy-dense snacks were captured to a slightly less extent than reported in the HEI. In almost all cases, it was not possible to determine the sugar content of drinks. It was possible to identify milk type using the color of the bottle tops. The presence of satellite TV was rarely captured, and child snacking while watching TV was captured less frequently than reported in the HEI. In total, 4470 images (6%) were classified as uncodeable. Figure 1 shows some sample images of home environment features.

Validity estimates for the 42 home food environment variables are shown in Table 3. Of the 42 variables, 25 (60%) had satisfactory validity (ICC or kappa ≥0.40; percent agreement ≥80 where kappa could not be calculated). Validity estimates were good for fresh fruit, fresh vegetable, and full-fat milk availability, the variety of fresh vegetables, the display of food and drink (except sweet snacks), eating meals as a family, and child eating lunch/dinner while watching TV. Particularly low validity estimates were reported for tinned and frozen vegetable availability and variety, and sweet snack availability. For the display of confectionery, percent agreement was high (87%), but kappa was –0.07 because there was just one yes response at the time of the HEI and one yes response captured by SenseCam (ppos was 0.00, but pneg was 0.93).

Validity estimates for the home activity and media environment variables are also shown in Table 3. The presence of a garden and play equipment had good validity (kappa >0.60). Of the 10 home media environment variables, 9 (90%) had satisfactory validity and 5 (50%) had good or excellent validity. Validity was lower for the number of household computers (ICC 0.3).

### Semistructured Interview

All but 1 participant completed the semistructured interview. All completing participants said that the SenseCam was straightforward to use. Initially, 1 participant had trouble charging the camera, and 2 forgot to charge it. Two participants said that the camera sometimes got in the way when they carried their children. Another suggested using a smaller, more discreet camera.

A total of 7 participants said that they forgot to wear the camera on some occasions: when they were returning from an outing, rushing in the morning to get ready for work, or when their children were not around. Situations where participants said they chose not to wear the SenseCam included trips to the bathroom, getting their children ready for bed, and when they had a visitor.

Almost all participants said that wearing the SenseCam made them think about aspects of their behavior and household routines. For example, one of the participants felt that their children were not eating healthily, watched too much TV, and needed to do more constructive activities. Although participants reported that they were aware of their behavior, most said that wearing the camera did not modify it. Two participants said that wearing the camera did affect their behavior: 1 said that they made more of an effort to eat with their child, and the other said that they tried to have meals at the table instead of while watching TV.

Participants generally reported that they were less aware of the camera as time went on. All participants reported that their children were interested in the camera, although this lessened with time. One participant said that their child was initially shy around the camera, and 1 thought that their children behaved better than usual.

Overall, participants were generally positive about the camera. A third of the participants said that they would be happy to wear the camera for a longer period of 1 to 2 weeks; the remaining participants felt that 4 days was sufficient. All participants felt that the SenseCam may be helpful to families that need to change aspects of their behavior or household routine.

**Table 3:** Validity estimates for the home environment features (N=15).

Home environment feature		Intraclass correlations (95% CI)	Kappa (95% CI)	% Agreement
**Food availability**				
	Fresh fruit^a^	—	—^b^	100
	Tinned fruit	—	—^b^	60
	Dried fruit	—	0.39 (0.06 to 0.72)	67
	Frozen fruit^a^	—	—^b^	80
	Fresh vegetables^a^	—	—^b^	93
	Tinned vegetables	—	0.12 (–0.11 to 0.35)	53
	Frozen vegetables	—	0.11 (–0.09 to 0.30)	40
	Savory snacks^a^	—	0.45 (0.04 to 0.87)	73
	Sweet snacks	—	0.13 (–0.07 to 0.32)	33
	Confectionery	—	0.31 (–0.07 to 0.69)	60
	Fruit juice^a^	—	0.59 (0.16 to 1.01)	80
	Squash^a^	—	0.51 (0.06 to 0.97)	80
	Fizzy drinks	—	0.19 (–0.35 to 0.72)	73
	Smoothies^a^	—	0.44 (–0.17 to 1.06)	87
	Skimmed/semi-skimmed milk^a^	—	0.47 (0.07 to 0.88)	80
	Full-fat milk^a^	—	0.73 (0.41 to 1.04)	87
**Food variety**				
	Fresh fruit^a^	0.43 (–0.09 to 0.76)	—	—
	Tinned fruit	—^b^	—	—
	Dried fruit	0.19 (–0.34 to 0.63)	—	—
	Frozen fruit	—^b^	—	—
	Fresh vegetables^a^	0.72 (0.35 – 0.90)	—	—
	Tinned vegetables	0.28 (–0.25 to 0.68)	—	—
	Frozen vegetables	0.00 (–0.49 to 0.50)	—	—
	Savory snacks	0.37 (–0.15 to 0.73)	—	—
	Sweet snacks^a^	0.46 (–0.04 to 0.78)	—	—
	Confectionery	0.38 (–0.14 to 0.74)	—	—
**Food displayed**				
	Any fruit^a^	—	—^b^	93
	Ready-to-eat vegetables^a^	—	—^b^	87
	Savory snacks^a^	—	—^b^	100
	Sweet snacks	—	–0.19 (–0.40 to 0.02)	67
	Confectionery^a^	—	–0.07 (–0.19 to 0.05)^c^	87
	Fruit juice^a^	—	—^b^	100
	Squash^a^	—	0.76 (0.26 to 1.26)	93
	Fizzy drinks^a^	—	—^b^	93
	Smoothies^a^	—	—^b^	100
**Family meals**				
	Breakfast^a^	—	—^b^	100
	Lunch^a^	—	—^b^	83
	Dinner^a^	—	0.63 (–0.09 to 1.35)	92
**Child eating while watching TV**				
	Breakfast	—	—^b^	77
	Lunch^a^	—	—^b^	92
	Dinner^a^	—	0.63 (–0.16 to 1.41)	92
	Snacks	—	0.10 (–0.36 to 0.57)	64
**Activity facilities**				
	Garden^a^	—	0.67 (0.26 to 1.07)	87
	Garden equipment^a^	—	0.63 (–0.03 to 1.28)	92
**Household media equipment**				
	Number of TVs	0.97 (0.92 to 0.99)	—	—
	Number of VCR/DVD players^a^	0.82 (0.55 to 0.94)	—	—
	Number of computers	0.30 (–0.23 to 0.69)	—	—
	Number of games consoles^a^	0.55 (0.08 to 0.82)	—	—
	Presence of cable or satellite^a^	—	—^b^	100
**Child’s bedroom media equipment**				
	TV^a^	—	0.76 (0.27 to 1.25)	93
	Computer^a^	—	—^b^	100
	Console^a^	—	0.63 (–0.06 to 1.33)	93
**Caregiver TV viewing (hours)**				
	Weekday^a^	0.55 (–0.13 to 0.88)	—	—
	Weekend^a^	0.57 (–0.15 to 0.90)	—	—

^a^Feature has satisfactory validity.

^b^ICC was not calculated due to zero variance items or kappa could not be calculated due to cell counts equalling zero.

^c^There was just one yes response at the time of the HEI and one yes response captured by SenseCam (ppos was 0.00, but pneg was high [0.93]).

## Discussion

### Principal Findings

This study investigated whether a wearable camera can be used to examine the early obesogenic home environment and whether it is useful for validation purposes. The SenseCam captured all 54 home environment features but with varying detail. Features that were captured less frequently included tinned and frozen foods, sweet snacks, and satellite TV. It was not possible to fully capture mealtime and TV viewing behaviors due to there being a single wearer and a limited wearing period. Validity estimates were at least satisfactory for two-thirds of the home environment features. Lower agreement was reported for food variety (except for fresh vegetables) and the number of computers in the home. The SenseCam was generally acceptable to participants, although there were reservations.

While the findings indicate that the SenseCam can be used to examine the obesogenic home environment, a primary issue is that what is captured depends on the actions of the wearer. Although this highlights the utility of the SenseCam as a behavioral measure, it also meant that it was often not possible to determine whether the SenseCam missed a particular feature or whether the feature truly was absent. For most cases of disagreement, a feature was reported at the time of the HEI but not captured by the SenseCam. This was particularly the case for tinned and frozen foods, sweet snacks, and media equipment (excluding TVs). It is possible that certain foods and media equipment were available in the home during the wearing period even though they were not captured.

### Comparison With Prior Work

Bryant and colleagues [7] reported generally moderate to high agreement when using home visits to validate their Healthy Home Survey. Overall agreement was high for the presence of all food types, suggesting that the low agreement for some food types in our study may indeed have been due to the SenseCam missing this information. Agreement for food variety was also higher than reported in our study. However, lower values (ICCs) were reported for sweet (0.30) and savory (0.48) snack variety in their study, suggesting that some discrepancies in our study may be due to other reasons than the SenseCam missing information, such as natural changes in food availability. As in our study, agreement for the presence of a garden and play equipment was high. For the number of computers and game consoles, agreement was higher than in our study (65% and 73%, respectively). However, in our study, it was possible to capture eating and TV viewing behavior, with acceptable agreement given the limited wearing period.

There were some cases of disagreement where a feature was not reported in the HEI but was captured by the SenseCam. For example, 2 participants did not report fizzy drinks, but these were present during the wearing period. It is feasible that these differences were due to natural changes in food availability; however, it could also reflect some bias in responding during the HEI. Previous research comparing self-reports to SenseCam images have found that individuals may overestimate their activity levels [19] and underestimate their dietary intake [21]. To determine whether differences really were due to changes in food availability, it would have been useful to ask participants about their shopping habits during the wearing period.

It is noteworthy that the SenseCam captured fewer sweet snacks than were reported in the HEI while slightly more fresh fruit and vegetables were captured. Although this could be a chance finding, participants may have modified their access to certain foods in the home. However, it is not clear if any behavioral effect would result from wearing the camera, completing the interview, or both. A larger scale validation study could use counter-balancing to control for any potential order effects. Nevertheless, most participants said that although wearing the camera made them reflect about their home environment, they did not think that it affected their behavior. When behavior is habitual, behavioral responses are activated automatically [30].

### Limitations

The large amount of data accumulated by the SenseCam is important to consider. Manual coding is time-consuming and errors can occur, although interrater reliability in this study was high. Automatic coding procedures for the home environment are needed, particularly if research uses longer wearing periods and involves multiple family members.

Another factor to consider is participant recruitment, as many families contacted in this study were not comfortable with the idea of wearing the camera. The families contacted had previously taken part in a survey-based study; therefore, although they agreed to be contacted for future studies, they may have been happy to take part only in other survey-based research. The sample size was small and comprised mainly white and university-educated participants, which limits our ability to generalize the findings.

### Implications and Recommendations

Taken together, the findings suggest that the SenseCam may be particularly useful for assessing behavioral aspects of the home environment and understanding how individuals interact with their home environment more generally, while home visits may be needed to more rigorously assess the availability of food and media equipment. A future study could directly compare SenseCam images with the results of home visits.

Having a longer wearing period or having multiple family members wear a SenseCam might provide a more comprehensive picture of the home environment. Most participants felt that 4 days was sufficient, so some form of incentive might be needed for a longer wearing period. Offering an incentive may also encourage less motivated, harder-to-reach families to take part in future studies, and it may minimize data loss if participants are motivated to keep the camera on for longer. In this study, participants were asked to remove the camera whenever they went outside of the home environment to minimize the chance of certain ethical issues arising and because it wasn’t necessary for participants to wear the camera outside. However, future research could have participants wear the camera outside of the home environment, as previous research has done [19,20], provided that certain ethical issues are taken into consideration. The SenseCam was considered unsuitable for young children to wear, although older children could wear one.

Using a device that can capture higher quality images would also benefit future research. Since the start of this study, the SenseCam has been superseded with newer models that can capture indoor images to a higher standard. Asking participants to clarify certain images may also help to minimize data loss.

### Conclusions

This study found that a wearable camera can be used to examine and validate aspects of the obesogenic home environment. While the SenseCam can capture physical aspects of the home environment such as food availability, its added strength is in capturing behavior. An optimal validation procedure could use a combination of home visits and wearable cameras.

## Appendix

Multimedia Appendix 1 Home environment features as assessed in the Home Environment Interview.

## Abbreviations

HEI: Home Environment Interview
ICC: intraclass correlation coefficient
pneg: proportion of negative agreement
ppos: proportion of positive agreement
