# Supplementary material for: Exploring the Potential of a Wearable Camera to Examine the Early Obesogenic Home Environment: Comparison of SenseCam Images to the Home Environment Interview
Source: J Med Internet Res. 2017 Oct 12;19(10):e332. doi: 10.2196/jmir.7748 (PMC5658644; doi:10.2196/jmir.7748)
Supplement: Multimedia Appendix 1 [file jmir_v19i10e332_app1.pdf]

Multimedia Appendix 1. Home environment features as assessed in the Home Environment Interview

Home environment feature	Corresponding HEI question
**Food availability**	
Fresh fruit	*Do you have any fresh fruit in your home now?*
Tinned fruit	*Do you have any tinned or jarred fruit in your home now?*
Dried fruit	*Do you have any dried fruit, such as raisins, dried apricots, or dates in your home now?*
Frozen fruit	*Do you have any frozen fruit in your home now?*
Fresh vegetables	*Do you have any fresh vegetables in your home now? This includes salad items such as lettuce, cucumber, and tomato but not potatoes.*
Tinned vegetables	*Do you have any tinned or jarred vegetables for example tinned tomatoes, sweetcorn, or jarred beetroot, in your home now? This includes tinned pulses such as chickpeas, kidney beans and lentils.*
Frozen vegetables	*Do you have any frozen vegetables in your home now?*
Savory snacks	*Do you have any savory snacks for example peanuts, crisps, tortillas and cheesy biscuits in your home now?*
Sweet snacks	*Do you have any sweet snacks for example cakes, biscuits or ice-cream in your home now?*
Confectionery	*Do you have any confectionery in your home now? This includes sweets and chocolate.*

Home environment feature	Corresponding HEI question
Fruit juice<br>Squash<br>Fizzy drinks<br>Smoothies<br>Skimmed/semi-skimmed milk<br>Full-fat milk	*Do you have any non-alcoholic drinks other than water in your home now? Examples are fruit juice, squash, fizzy pop, ready-made fruit flavored drinks, smoothies, and milk.*
**Food variety**	
Fresh fruit	*What types of fresh fruit do you have in your home now? Have you remembered fresh fruit in your fridge, in a fruit bowl and in your cupboards?*
Tinned fruit	*What types of tinned or jarred fruit do you have in your home now? Have you remembered tinned or jarred fruit in your fridge and in your cupboards?*
Dried fruit	*What types of dried fruit do you have in your home now? Have you remembered dried fruit in a fruit bowl and in your cupboards?*
Frozen fruit	*What types of frozen fruit do you have in your home now?*
Fresh vegetables	*What types of fresh vegetables do you have in your home now? Have you remembered fresh vegetables in your fridge and in your cupboards?*
Tinned vegetables	*What types of tinned or jarred vegetables do you have in your home now? Have you remembered tinned or jarred vegetables in your fridge and in your cupboards?*
Frozen vegetables	*What types of frozen vegetables do you have in your home now?*

Home environment feature	Corresponding HEI question
Savory snacks	*What types of savory snacks do you have in your home now? Snacks like plain rice cakes, oatcakes, and breadsticks are not included. Have you remembered savory snacks in your fridge and in your cupboards?*
Sweet snacks	*What types of sweet snacks do you have in your home now? Do not include sweets or chocolate. Have you remembered sweet snacks in your fridge and in your cupboards?*
**Confectionery**	*What types of confectionery do you have in your home now? Have you remembered confectionery in your fridge, in a bowl and in your cupboards?*
**Food displayed**	
Any fruit	*Without opening any fridge or cupboard doors, is there any kind of fruit in your home now; displayed out in the open?*
Ready-to-eat vegetables	*Do you have any ready to eat fresh vegetables on a shelf in the fridge or on the kitchen counter now? These include baby carrots, cherry tomatoes, or vegetables that you have sliced to make them ready to eat.*
Savory snacks	*Without opening any fridge or cupboard doors, are there any kind of savory snacks in your home now; displayed out in the open?*
Sweet snacks	*Without opening any fridge or cupboard doors, are there any kind of sweet snacks in your home now displayed out in the open?*

Home environment feature	Corresponding HEI question
Confectionery	*Without opening any fridge or cupboard doors, is there any kind of confectionery in your home now displayed out in the open?*
Fruit juice<br>Squash<br>Fizzy drinks<br>Smoothies	*Without opening any fridge or cupboard doors, are there any non-alcoholic drinks in your home now; displayed out in the open? What types of non-alcoholic drinks are displayed out in the open?*
**Family meals**	
Breakfast	*How many days a week do your family sit at a table to eat breakfast together? This includes occasions when it is just <child's name> and yourself or just <child's name> and your <husband/wife/partner>. Only include occasions where you or your <husband/wife/partner> actually eat with your child.*
Lunch	*How many days a week do your family sit at a table to eat a midday meal together? This includes occasions when it is just <child's name> and yourself or just <child's name> and your <husband/wife/partner>. Only include occasions where you or your <husband/wife/partner> actually eat with your child.*
Dinner	*How many days a week do your family sit at a table to eat an evening meal together? This includes occasions when it is just <child's name> and yourself or just <child's name> and your <husband/wife/partner>. Only include occasions where you or your <husband/wife/partner> actually eat with your child.*

Home environment feature	Corresponding HEI question
**Child eating while watching TV**	
Breakfast	*How many days per week does <child's name> eat breakfast while watching TV?*
Lunch	*How many days per week does <child's name> eat a midday meal while watching TV?*
Dinner	*How many days per week does <child's name> eat an evening meal while watching TV?*
Snacks	*How many days per week does <child's name> eat snacks while watching TV?*
**Activity facilities**	
Garden	*Do you have a garden or outdoor space that <child's name> can play in?*
Garden equipment	*Do you have any usable play equipment such as swings, slides, climbing frames, trampolines in your garden (or outdoor space)?*
**Household media equipment**	
Number of TVs	*How many working TV's do you have in your home?*
Number of VCR/DVD players	*How many working VCR or DVD players do you have in your home?*
Number of computers	*How many working computers or laptops do you have in your home?*
Number of games consoles	*How many working games consoles, such as Play Station, Nintendo DS, Wii do you have in your home?*

Home environment feature	Corresponding HEI question
Presence of cable or satellite	*Do you have cable or satellite?*
**Child’s bedroom media equipment**	
TV	*Does <child’s name> have a working TV in their bedroom?*
Computer	*Does <child’s name> have a computer or laptop in his/her bedroom?*
Games console	*Does <child’s name> have a games console in their bedroom?*
**Caregiver TV viewing**	
Weekday	*On average, how long do you watch TV or DVDs during the following times of a typical weekday (Monday to Friday), at this time of year?*<br><br>*Morning (6am to 12 noon)*<br>*Afternoon (12am to 6pm)*<br>*Evening (6pm to midnight)*
Weekend	*On average, how long do you watch TV or DVDs during the following times of a typical weekend day, at this time of year?*<br><br>*Morning (6am to 12 noon)*<br>*Afternoon (12am to 6pm)*<br>*Evening (6pm to midnight)*

HEI = Home Environment Interview
